# Long-term survival after surgical resection for recurrent hepatic and pulmonary metastases of intrahepatic cholangiocarcinoma: a case report

**DOI:** 10.1186/s40792-019-0693-7

**Published:** 2019-08-20

**Authors:** Mihoko Yamada, Atsuki Arimoto, Yoshitaka Toyoda, Shinya Watanabe, Keiji Aizu, Fumiya Sato, Akinori Fujieda, Ryuzo Yamaguchi

**Affiliations:** Division of Surgery, Kasugai Municipal Hospital, Aichi, Japan

**Keywords:** Intrahepatic cholangiocarcinoma, Pulmonary resection, Repeat hepatectomy

## Abstract

**Background:**

A few reports to date have described the effectiveness of surgical resection for recurrent intrahepatic cholangiocarcinoma (ICC). We report in this study a patient who achieved long-term survival after surgical resection for recurrent hepatic and pulmonary metastases of ICC.

**Case presentation:**

A 62-year-old man was referred to our hospital for examination of a tumor in the left lobe of the liver. Computed tomography (CT) scans of the abdomen revealed a hypovascularized tumor, 30 mm in hepatic segment 2 (S2). The patient was diagnosed with a mass-forming type of ICC. A left lateral sectionectomy with regional lymph node dissection was performed. Histopathological examination showed moderately differentiated adenocarcinoma in the hepatic S2 with lymph node metastasis. There were two intrahepatic metastases around the main tumor. The pathological stage of the ICC was pT2pN1M0pStageIIIB. The patient did not receive adjuvant chemotherapy after surgery. Twelve months after surgery, liver lesions in S4/S8 and S7 were detected on CT scans. A partial hepatectomy was performed. The histopathological features were similar to those of the previous ICC. The patient did not receive adjuvant chemotherapy after the repeat hepatectomy. Four years and four months after this repeat hepatectomy, CT scans showed multiple nodes in S4 and S10 of the left lung and in S1 of the right lung. Wedge resection of the left upper lobe and sectionectomy in S10 of the left lung were performed. Histopathological findings of the resected lung nodules were compatible with metastatic ICC. The nodule in S1 of the right lung was too small to be diagnosed as metastasis; therefore, it was not resected. After pulmonary resection, the patient was treated with gemcitabine and cisplatin for 6 months. After chemotherapy, the size of the nodule in S1 increased gradually. One year and ten months after the pulmonary resection, we performed wedge resection of S1 of the right lung, and the histopathological findings were compatible with metastatic ICC. The patient is alive without evidence of disease 8 years after the initial surgery and 8 months after the last pulmonary resection.

**Conclusions:**

ICC with poor prognostic factors can frequently recur; however, surgical resection for recurrent ICC might, for selected patients, enable long-term survival.

## Background

Intrahepatic cholangiocarcinoma (ICC) has a poor prognosis. Surgical resection, which includes hepatectomy and regional lymph node dissection, is the only established curative therapy [1]. Although curative resection is performed, the majority of patients develop ICC recurrence, especially intrahepatic recurrence. Several retrospective studies have documented that repeat hepatectomy for recurrent ICC provides survival benefits [2, 3]. This procedure has, therefore, been performed aggressively. Regarding pulmonary recurrence, there are a few reports that have documented the survival benefits of surgical resection for recurrent ICC. However, case reports of pulmonary metastasectomy are more limited than those of repeat hepatectomy. We report here a patient having undergone repeat hepatectomy and pulmonary resection with long-term survival.

## Case presentation

**Fig. 1:**
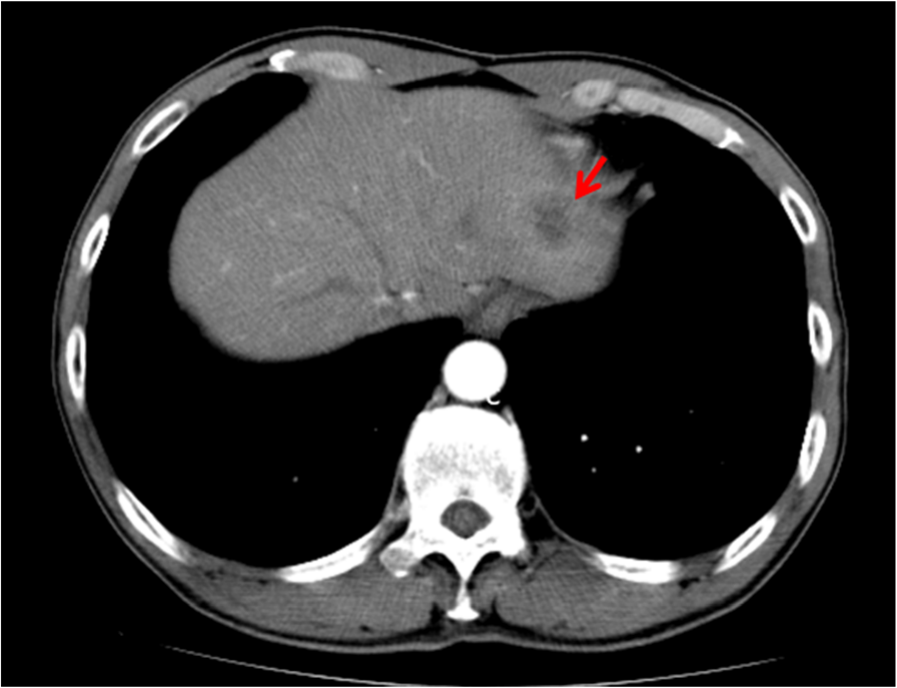
CT scan shows a hypovascularized tumor, measuring 30 mm, in hepatic S2 (arrow)

**Fig. 2:**
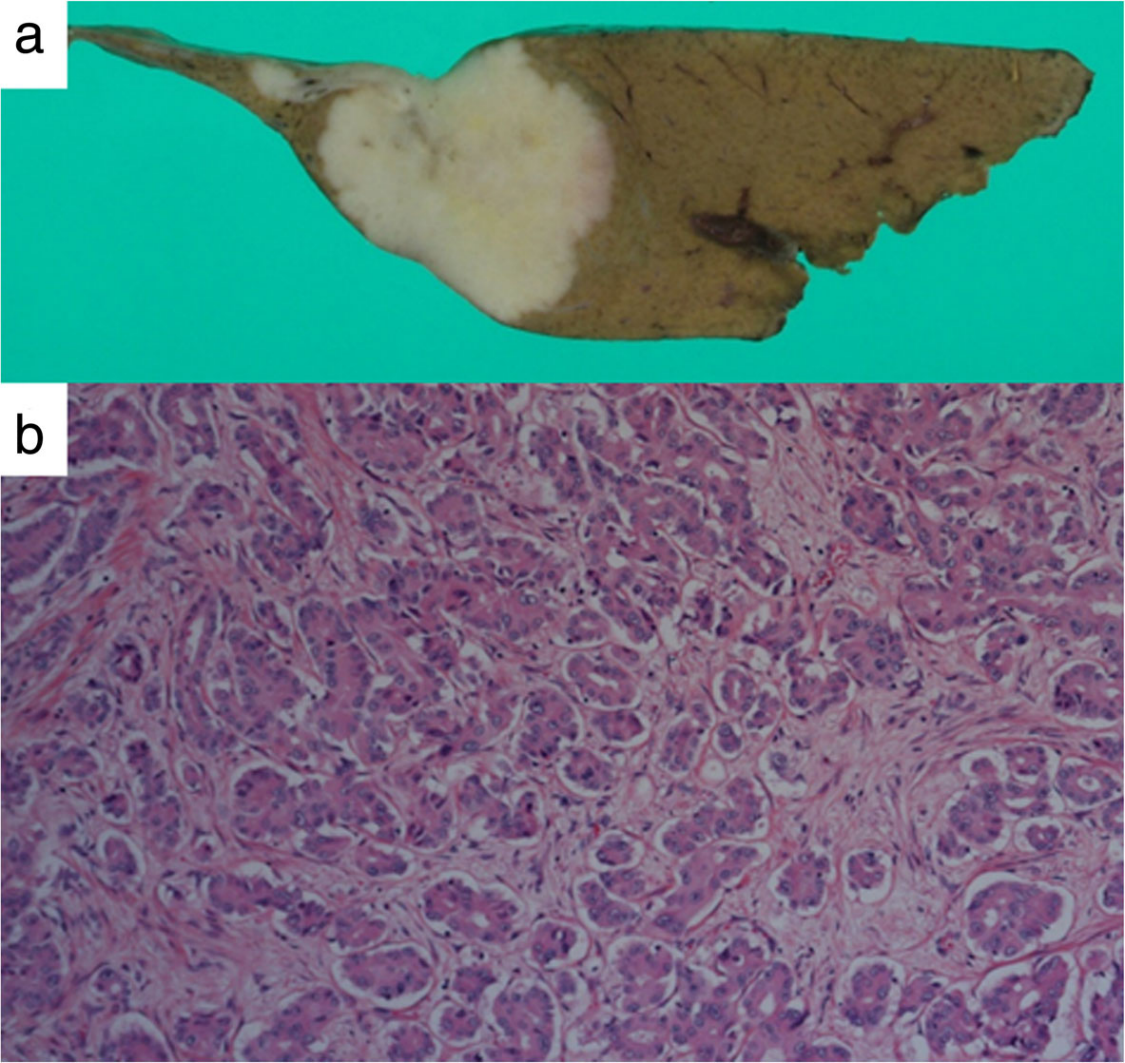
**a** The cut surface of the resected specimen shows a gray-white nodular lesion. **b** Microscopic finding shows moderately differentiated adenocarcinoma in S2 of the liver

A 62-year-old man was referred to our hospital for examination of a liver tumor in the hepatic left lobe. He had diabetes mellitus and prior hepatitis B virus infection. Laboratory test results for carcinoembryonic antigen (CEA) and carbohydrate antigen 19-9 (CA 19-9) were unremarkable. Computed tomography (CT) scans of the abdomen and gadoxetic acid-enhanced magnetic resonance imaging (EOB-MRI) revealed a hypovascularized tumor, measuring 30 mm in hepatic segment 2 (S2), and no enlarged regional lymph nodes (Fig. 1). A liver biopsy was performed to analyze the tumor. A histopathological examination showed adenocarcinoma. On immunohistochemistry, the carcinoma cells were positive for cytokeratin 7 (CK7), CA 19-9, and EMA, and negative for CK20, α-fetoprotein, and thyroid transcription factor-1 (TTF-1). The patient was diagnosed with a mass forming (MF) type of ICC. A left lateral sectionectomy, with regional lymph node dissection along the proper, left and middle hepatic arteries and the upper branch of the left gastric artery, was performed. Histopathological examination showed moderately differentiated adenocarcinoma in hepatic S2, with one lymph node metastasis around the portal vein in the hepatoduodenal ligament and the minor branch of the portal vein invasion in the main tumor (Fig. 2); s0, n1, vp1, vv0, va0, and p0. There were two intrahepatic metastases in the same S2 around the main tumor. According to the eighth edition of the TNM staging system of the Union for International Cancer Control [4], the pathological stage of the ICC was pT2pN1M0pStageIIIB. The postoperative course was uneventful, and the patient was discharged on the tenth postoperative day. Although the adjuvant chemotherapy was recommended to him because there was a high possibility of recurrence of the carcinoma, he refused to undergo it.

**Fig. 3:**
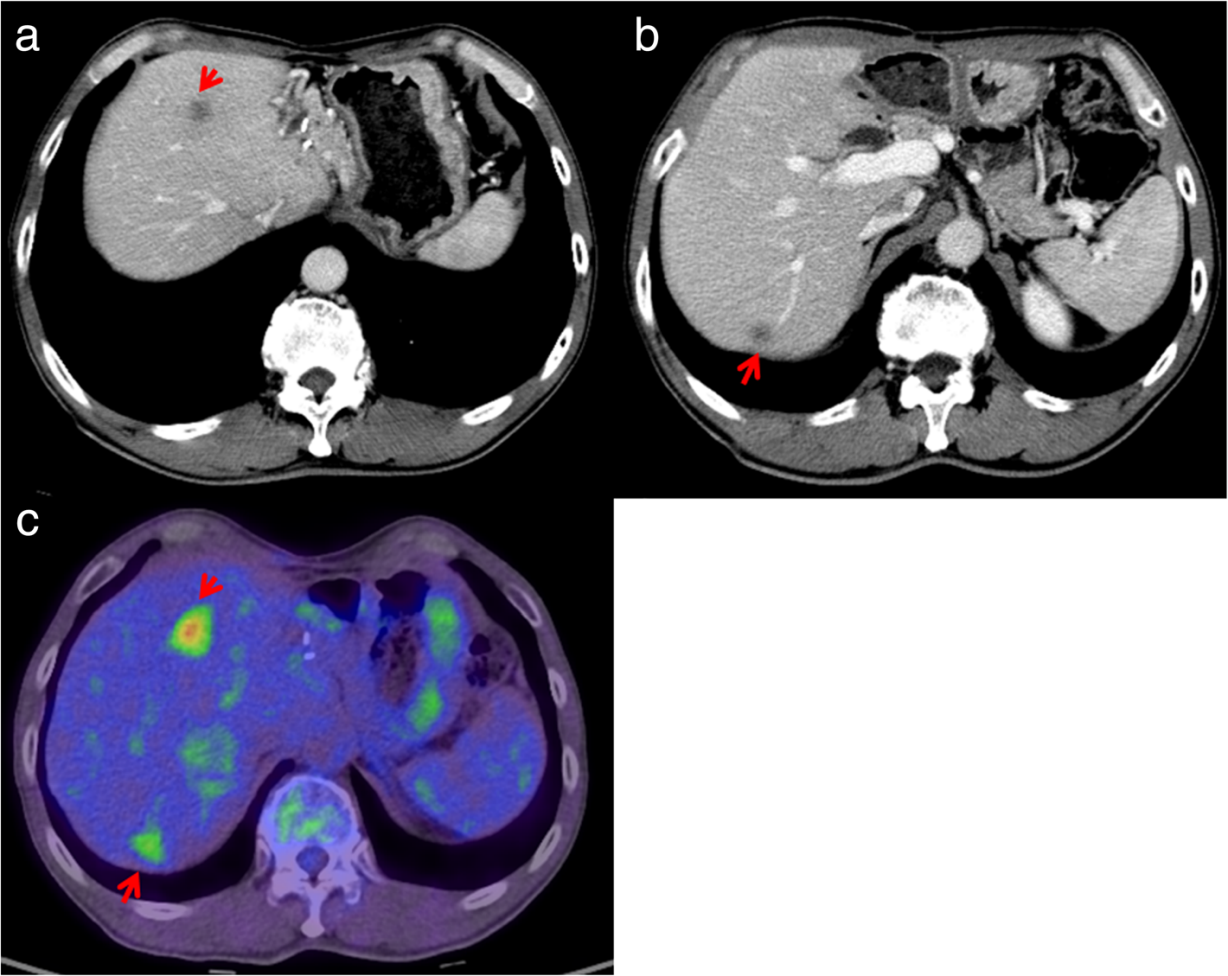
CT scan shows hypovascularization in hepatic S4/S8 (**a**) and S7 (**b**). **c** On PET-CT, abnormal FDG uptake is observed in both hepatic tumors (arrows)

**Fig. 4:**
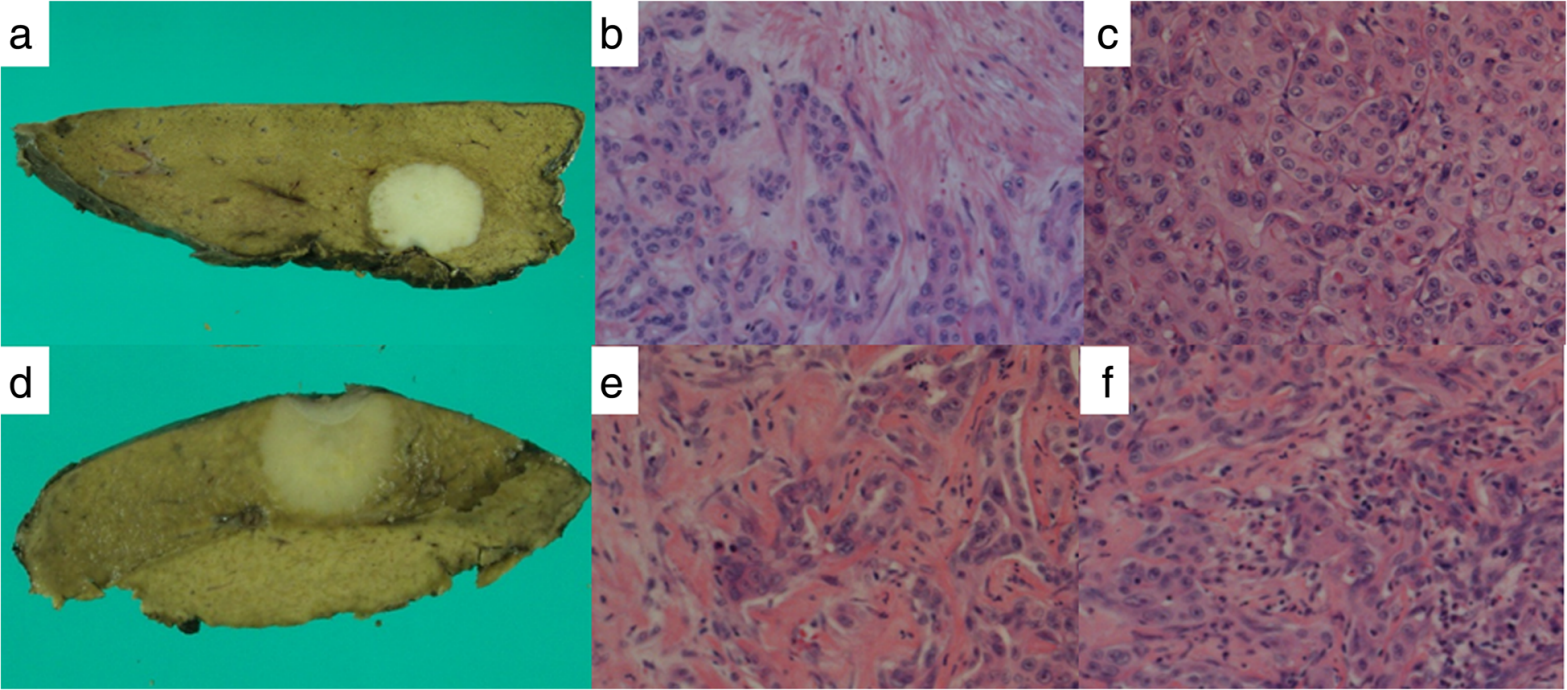
The cut surface of the resected specimen shows a gray-white nodular lesion in hepatic S4/S8 (**a**) and S7 (**d**). Microscopic findings show moderately differentiated adenocarcinoma in the center of the tumor (**b**, **e**) and poorly differentiated adenocarcinoma at the margin of the tumor (**c**, **f**). **b**, **c**, **e**, **f** Hematoxylin and eosin staining, × 100

**Fig. 5:**
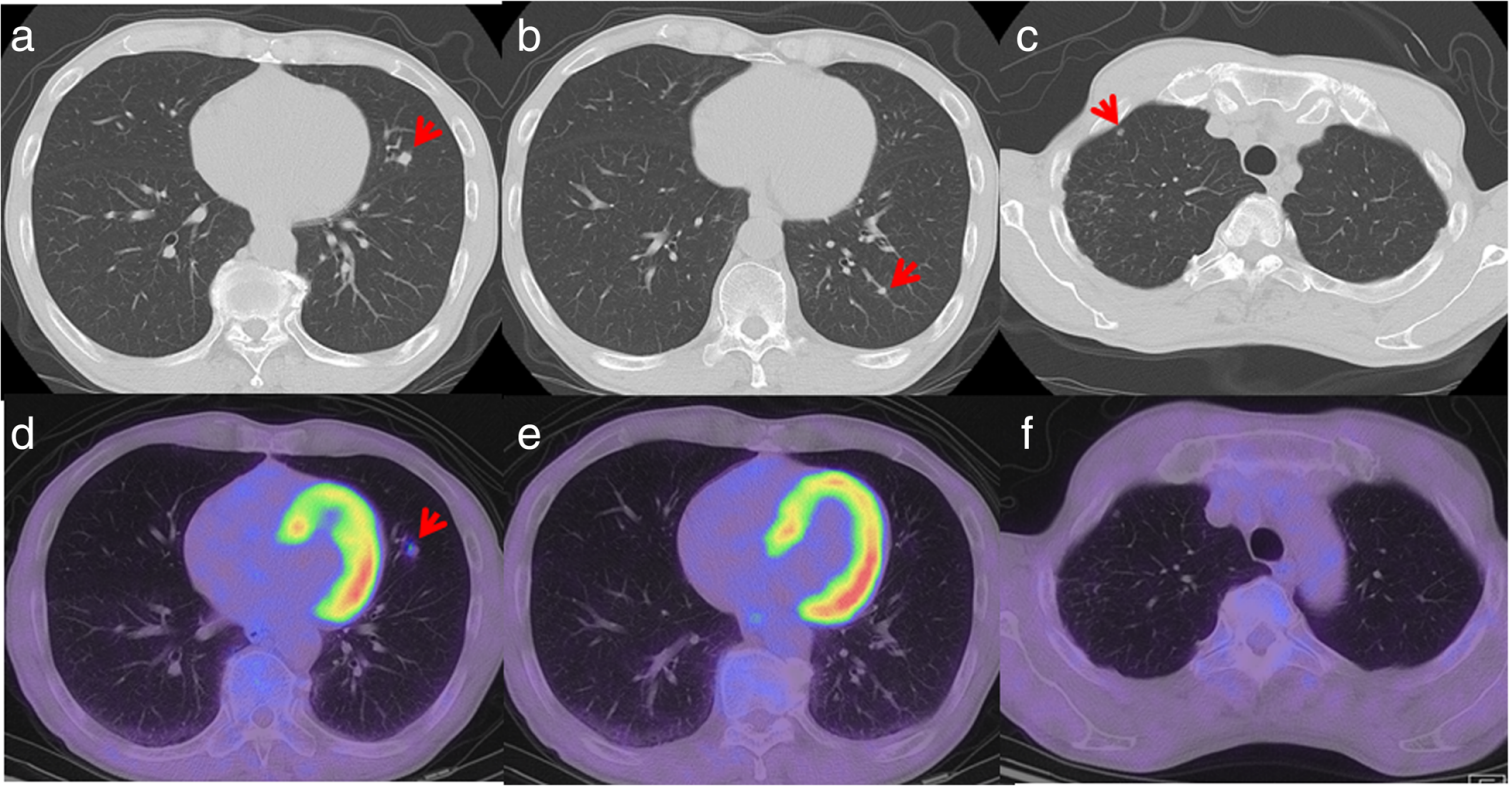
CT scan shows multiple nodes in S4 (**a**) and S10 (**b**) of the left lung and S1 (**c**) of the right lung. On PET-CT, abnormal FDG uptake is observed in S4 (**d**) of the left lung, but not in S10 (**e**) of the left lung or S1 (**f**) of the right lung (arrows)

**Fig. 6:**
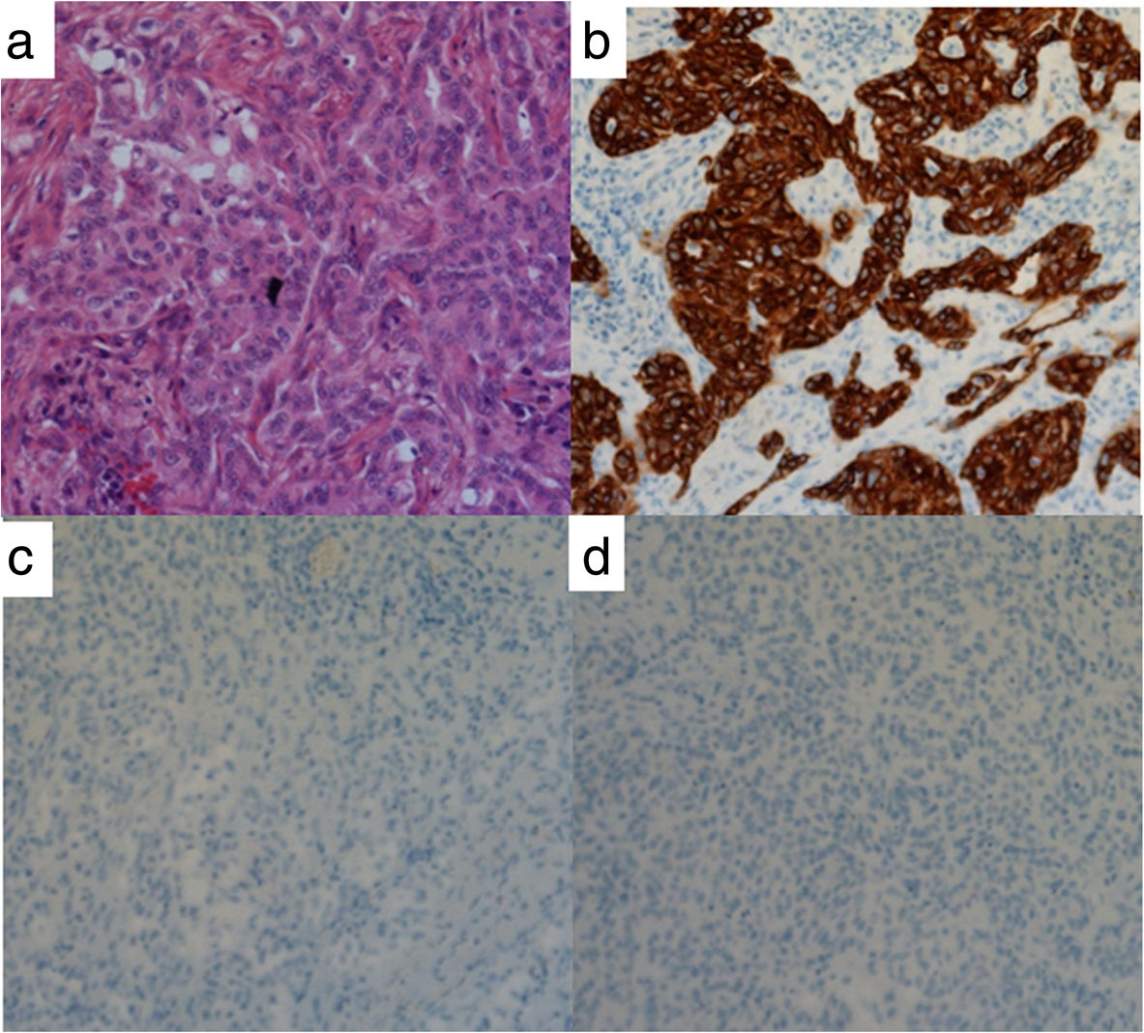
**a** Microscopic findings from the lung tumor of S4 show poorly differentiated adenocarcinoma. **b** CK 7 is positive. **c**, **d** CK 20 and TTF-1 are negative (Hematoxylin and eosin staining [× 100] (**a**), and immunohistochemical expression [× 100] of CK 7 (**b**), CK 20 (**c**), and TTF-1 (**d**))

Twelve months after surgery, liver lesions in S4/S8 and S7 were detected on CT scans (Fig. 3a, b). No other liver lesions were found using EOB-MRI. On positron emission tomography-computed tomography (PET-CT), abnormal fluorodeoxyglucose (FDG) uptake was observed only in hepatic tumors, and extrahepatic lesions were not detected (Fig. 3c). Neither CEA nor CA 19-9 was elevated. The patient wanted a second opinion on treatment other than surgery and chemotherapy. After observation for 3 months, the size of two recurrent liver tumors was slightly larger compared with that observed 3 months ago. However, without developing any other lesions, he underwent a partial hepatectomy for each lesion (Fig. 4a, d). A pathological examination of both resected tumors revealed moderately differentiated adenocarcinoma in the center of the tumors, which was similar to that of previous ICC (Fig. 4b, e). At the margin of the tumors, poorly differentiated adenocarcinoma was detected (Fig. 4c, f). On immunohistochemistry, the carcinoma cells were positive for CK 7 and negative for CK 20 and TTF-1. Histopathological features were similar to those of the previous ICC; therefore, the patient was diagnosed with recurrence of ICC. He was discharged on the seventh postoperative day. Although the adjuvant chemotherapy was repeatedly recommended to him, he refused to undergo the therapy after the repeat hepatectomy. Four years and four months after the repeat hepatectomy, CT scans showed multiple nodes in S4 and S10 of the left lung and in S1 of the right lung (Fig. 5a–c). On PET-CT, FDG uptake was observed only in S4 of the left lung (Fig. 5d–f). After observation for 3 months, the size and number of tumors did not change. Therefore, wedge resection of the left upper lobe and sectionectomy of S10 of the left lung were performed. The histopathological findings of the resected lung nodules were compatible with metastatic ICC (Fig. 6a). On immunohistochemistry, the carcinoma cells were positive for CK 7 and negative for CK 20 and TTF-1 (Fig. 6b–d). The nodule noted in S1 of the right lung was too small to be diagnosed for metastasis; therefore, it was not resected. After pulmonary resection, He was treated with gemcitabine (1000 mg/m^2^) and cisplatin (25 mg/m^2^) which were infused on day 1 and day 8. This regimen was repeated at 21-day intervals for 6 months. After chemotherapy, the size of the nodule in S1 of the right lung increased gradually. One year and ten months after the pulmonary resection, we confirmed that there were no other metastatic lesions and we performed a wedge resection of S1 of the right lung. Histopathological findings were compatible with metastatic ICC. On immunohistochemistry, the carcinoma cells were positive for CK 7 and negative for CK 20 and TTF-1. The patient is alive without evidence of disease 8 years after the initial surgery and 8 months after the last pulmonary resection.

## Discussion

The 5-year overall survival (OS) rate of ICC after curative intent surgery was reported to be only 15*–*40% [5]. The risk factors for ICC recurrence after surgery include multiple tumors, vascular invasion, and lymph node metastasis [1, 6]. The survival benefits of adjuvant chemotherapy and chemoradiotherapy remain controversial. Five randomized clinical studies of adjuvant chemotherapy in patients with biliary tract cancer are currently ongoing, and three studies have recently been published [7–9]. For intrahepatic cholangiocarcinoma, several retrospective studies have evaluated the effect of adjuvant chemotherapy. Specifically, Reames et al. [10] in their study reported that adjuvant therapy was associated with a survival benefit in patients with advanced T stage (T2/3/4) (5-year OS: 37% vs. 30%; *p* = 0.006), periductal growth type (PI), or PI + MF subtype (5-year OS: 37% vs. 8%; *p* < 0.001) and lymph node metastasis (5-year OS: 18.3% vs. 12%; *p* = 0.050). Miura et al. [11] similarly described a survival benefit of adjuvant chemotherapy in patients with N1 disease (median OS: 19.8 vs. 10.7 months; *p* < 0.001), T3/4 tumors (median OS: 21.3 vs. 15.6 months, *p* < 0.001), and R1/R2 surgical resection (median OS: 29.5 vs. 11.6 months, *p* = 0.006). Our patient had several risk factors, such as multiple intrahepatic lesions, lymph node metastases, and vascular invasion, for recurrence of ICC after surgery, and he was recommended for adjuvant chemotherapy. However, the patient rejected adjuvant chemotherapy and was carefully monitored.

The resection of recurrent ICC demands challenging. Recently, repeat hepatectomy for recurrent ICC has been documented and has provided survival benefits [2, 3]. The median survival time after surgery for recurrent ICC was 10*–*66.6 months. Although the majority of the research has documented a small number of patients, Si et al. [3] in their study reported the survival outcome of 72 patients who underwent R0 repeat hepatic resection for recurrent ICC. The median time to recurrence was 13.6 months, and the median survival time was 45.1 months. The independent risk factors for overall survival were indicated as follows: recurrent tumor > 3 cm; multiple recurrent nodules; cirrhosis; and time to recurrence (TTR) < 1 year. In our patient, because he had risk factors of poor prognosis, such as multiple recurrent tumors and short TTR, he was observed for 3 months before repeat hepatectomy was performed and no other lesions were confirmed.

There have been a few reports to date of resection procedures for recurrent ICC, other than hepatectomy. Pulmonary resection for recurrent ICC has been described in several case reports [12], and there have additionally been few cases in small-sample studies published in English [13–15] (Table 1). There were eight cases with pulmonary resection for recurrent ICC, including seven reported cases and the present case. Two cases, including ours, had repeat pulmonary resection. The initial disease-free survival period was over 12 months in all patient cases except for one case, and survival period after recurrence was more than 60 months in five patient cases. The author previously reported that surgery for pulmonary metastases from cholangiocarcinoma should be considered in patients with a longer initial disease-free interval [16]. In our patient, repeat pulmonary resection for recurrent ICC was performed after a sufficient observation period. In particular, the slow-growing lesion of S1 of the right lung was resected 1 year and 10 months after it was identified, with confirmation of no other additional lesions.

**Table 1:** Reported cases of patients who underwent pulmonary resection for recurrent intrahepatic cholangiocarcinoma

				Primary tumor						1st recurrence		2nd recurrence		3rd recurrence		4th recurrence				
Case	Author/Year	Age (y)	Sex	MT	Size of tumor (mm)	Number of tumor	Histology	V	LN	Site	Duration (month)	Site	Duration (month)	Site	Duration (month)	Site	Duration (month)	DFS (month)	Survival (month)	Outcome
1	Morise [12]/2008	57	F	NR	NR	Multiple	mod	P	P	Liver	3	Lung	32	Liver	46	Liver	59	3	62	AWD
2	Saiura [13]/2011	44	M	MF	80	NR	mod	P	A	Lung	74	Adrenal	88	Lung	107	Lung	137	74	63	NED
3		67	M	MF	50	NR	mod	P	P	Liver	13	Lung	44					13	117	NED
4	Park [14]/2016	NR	NR	NR	NR	NR	NR	NR	NR	Lung	54							54	61	NED
5	Ohira [15]/2018	40	M	MF	20	Solitary	mod	P	A	Lung	44.4							44.4	34.7	NED
6		64	M	MF	20	Solitary	mod	P	A	Lung	64.8							64.8	37.2	DFD
7	Our case	62	M	MF	30	Multiple	mod	P	P	Liver	12	Lung	52	Lung	74			12	84	NED

*MT* macroscopic type, *V* vascular invasion, *LN* lymph node metastasis, *Duration* duration after initial surgery, *DFS* initial disease free survival, *Survival* survival after recurrence, *NR* not reported, *MF* mass forming type, *mod* moderately differentiated adenocarcinoma, *P* present, *A* absent, *AWD* alive with disease, *NED* no evidence of disease, *DFD* died from disease

## Conclusions

Our patient had several poor prognostic factors, and he did not receive adjuvant chemotherapy after the initial surgery. By performing repeat hepatectomy and pulmonary resection for recurrent ICC at the appropriate time after careful follow-up, a favorable outcome was conferred on the patient. Surgical resection for recurrent ICC might contribute toward long-term survival.

## Data Availability

Data sharing is not applicable to this article, as no datasets were generated or analyzed during the current study.

## Abbreviations

CA 19-9: Carbohydrate antigen 19-9
CEA: Carcinoembryonic antigen
CK: Cytokeratin
CT: Computed tomography
EOB-MRI: Gadoxetic acid-enhanced magnetic resonance imaging
FDG: Fluorodeoxyglucose
ICC: Intrahepatic cholangiocarcinoma
MF: Mass forming type
OS: Overall survival
PET-CT: Positron emission tomography-computed tomography
PI: Periductal growth type
S2: Segment 2
TTF-1: Thyroid transcription factor-1
TTR: Time to recurrence
